# The association between blood glucose levels and lipids or lipid ratios in type 2 diabetes patients: A cross-sectional study

**DOI:** 10.3389/fendo.2022.969080

**Published:** 2022-09-06

**Authors:** Liqun Wang, Ning Yan, Min Zhang, Ruiping Pan, Yuqi Dang, Yang Niu

**Affiliations:** ^1^ Ningxia Regional Key Laboratory of Integrated Traditional Chinese and Western Medicine for Prevention and Treatment of Regional High Incidence Disease, Ningxia Medical University, Yinchuan, China; ^2^ Key Laboratory of the Ningxia Ethnomedicine Modernization, Ministry of Education, Ningxia Medical University, Yinchuan, China; ^3^ Department of Epidemiology and Statistics, School of Public Health and Management at Ningxia Medical University, Yinchuan, China; ^4^ Key Laboratory of Environmental Factors and Chronic Disease Control, Ningxia Medical University, Yinchuan, China; ^5^ Heart Centre & Department of Cardiovascular Diseases, General Hospital of Ningxia Medical University, Yinchuan, China; ^6^ Department of Rehabilitation Medicine, General Hospital of Ningxia Medical University, Yinchuan, China; ^7^ Department of Chinese Medicine, The Second People’s Hospital of Shizuishan, Shizuishan, China; ^8^ Department of Endocrinology, Yinchuan Hospital of Traditional Chinese Medicine, Yinchuan, China

**Keywords:** blood glucose levels, type 2 diabetes mellitus, cross-sectional study, lipids, lipid ratios

## Abstract

**Background:**

Lipids and lipid ratios are associated with complications of diabetes mellitus type 2 (T2DM), such as cardiovascular disease, but the relationship between blood glucose levels and lipid or lipid ratios is not fully understood in T2DM patients. This study assesses the association between blood glucose levels and lipid or lipid ratios in a cohort of T2DM patients.

**Methods:**

A total of 1,747 Chinese T2DM patients from the Ningxia province of China were included in this cross-sectional study. Lipid parameters, including triglycerides (TG), total cholesterol (TC), high-density lipoprotein (HDL-C), low-density lipoprotein (LDL-C), and fasting blood glucose levels were measured quantitatively using standard methods. Fasting blood glucose was divided into three groups. A multiple mixed-effect linear regression model was conducted to identify a potential association between blood glucose and lipid parameters.

**Results:**

There was a positive association between blood glucose and TG levels (β=0.34, 95% *CI*: (0.20, 0.48), *p<*0.01); every 1 mmol/L increase in blood glucose levels resulted in a 0.34 mmol/L increase in TG. Blood glucose levels were also associated with high LDL (β=0.08, 95% *CI*: (0.02, 0.14), *p<*0.01), TG/HDL-C (β=0.31, 95% *CI*: (0.13, 0.49), *p<*0.01), and LDL-C/HDL-C (β=0.13, 95% *CI*: (0.06, 0.20), *p*<0.01) levels. After controlling for demographic variables, health-related behaviors, and physical health variables, a positive association between blood glucose levels and TG (β=0.31, 95% *CI*: (0.17, 0.45), *p<*0.01) and LDL-C (β=0.08, 95% *CI*: (0.02, 0.13), *p<*0.01) levels and an in increase in TG/HDL-C (β=0.28, 95% *CI*: (0.09, 0.46), *p<*0.01) and LDL-C/HDL-C (β=0.11, 95% *CI*: (0.04, 0.18), *p<*0.01) ratios was found.

**Conclusion:**

A correlation between blood glucose levels and serum lipids or lipid ratios has been established in this study. Blood glucose levels were positively associated with TG and LDL-C levels and elevated TG/HDL-C and LDL-C/HDL-C ratios.

## Introduction

Diabetes mellitus type 2 is a multisystem, chronic, noncommunicable disease that has reached epidemic proportions (1) and developed into a significant public health concern worldwide (2, 3). Both the case number and the prevalence of diabetes have risen over the past few decades, and it is predicted that the number of T2DM patients will increase to 700 million by 2045 (4). T2DM is a metabolic disease caused by the relative or absolute deficiency of insulin and is associated with elevated blood glucose levels (5). Elevated blood glucose is also linked with several other diseases, such as dyslipidemia (6). Dyslipidemia and diabetes are closely associated, hyperglycemia not only causes apoptosis of β-cells in the islets of Langerhans (glucotoxicity) but also determines the degree of accumulation of oxidized LDLs (7), and blood lipids can successfully treat the adverse outcomes of this disease (8). More than 75% of T2DM patients have mixed dyslipidemia that is characterized by low levels of high‐density lipoprotein cholesterol (HDL‐C) and high levels of triglyceride (TG) (9).

Increased serum concentrations of total cholesterol (TC), total triglycerides (TG), and low-density lipoprotein cholesterol (LDL-c), as well as low HDL-C, are considered lipid parameters that could predict the risk of coronary heart disease (10). There are several lipid ratio parameters that have been defined by prior studies, such as TC/HDL-C, TG/HDL-C, and LDL-C/HDL-C (11–13). T2DM is widespread worldwide and is a severe risk factor for cardiovascular disease (14). T2DM patients often present with characteristic plasma lipid and lipoprotein abnormalities, including low HDL-C, high LDL-C (15), and elevated TC levels (15). It is critical for clinicians to effectively manage dyslipidemia to reduce the incidence of cardiovascular disease in this patient population.

According to a previous study, high blood glucose levels were positively associated with having high TG and high TC (16). Blood lipid levels in patients with T2DM are affected by the degree of glycemic control (17). Wang et al. reported that fasting plasma glucose levels were significantly associated with HDL and TC but not with LDL and TG among T2DM patients in Qingdao, China (18). The glycemic control of patients with T2DM is also significantly associated with the visceral adiposity index; a high index indicates poor glycemic control (19). Controlling lipid profiles in patients with T2DM is essential to reducing mortality and complications (20). However, the association between blood glucose levels and lipids or lipids ratio parameters closely linked to obesity in Ningxia province, remains unknown. The current study explores the association between blood glucose levels and lipid or lipid ratio parameters in a cohort of T2DM patients from this geographic region. We hypothesized that blood glucose levels were associated with higher TG, TC, and LDL levels, higher TG/HDL‐C and LDL‐C/HDL‐C ratios, and lower HDL-C levels in T2DM patients.

## Methods

### Study design and patients

1,747 T2DM patients from 10 hospitals in Ningxia province, China, were enrolled in this population-based cross-sectional study from August 2019 to November 2020. Participants were recruited using PPS sampling. A related sampling procedure has been described formerly (21). The total sample size was evaluated by the formula: n = ${\text{Z}}_{\alpha /2}^{2}$ (1 - P)/ϵ2 P. According to a previous study, the prevalence of dyslipidemia in Chinese diabetes patients was 54.3% (22). We set P = as 0.543 in this study. If α was 0.05, Z_α/2_ was 1.96, and ϵ = 0.05, the calculated sample size was 1293. To prevent an invalid survey sample, we increased the sample by 20%, which made the minimum sample size in the survey 1551. The study was conducted during hospitalization. All participants over 18 years of age and had lived at their current address for a minimum of six months consented to participate in the study and agreed to complete a questionnaire and laboratory testing. Patients with severe mental disorders, a severe illness or language barrier that made communication impossible, pregnant or lactating women, diabetic ketoacidosis within the past month, malignant tumors, big surgery operation, or refusal to sign informed consent were excluded from the study. The Institutional Review Board of the Yinchuan Hospital of Traditional Chinese Medicine approved the study. Research procedures involving human participants conformed with the 1964 Helsinki declaration and its later amendments.

### Dependent variables

All participants underwent a thorough physical examination and provided blood samples for lipid testing, including fasting serum TC, TG, HDL‐C, and LDL‐C. Blood samples were taken from the T2DM patients, and the serum was separated. Then, serum samples were tested within two hours. FPG was measured using the hexokinase method. The enzymatic method was applied to test TG and TC. HDL-C and LDL-C were measured using the direct quantitation (peroxidase scavenging) method. Automatic biochemical detectors (SIEMENSADVIAXPT, Germany) were used to test those biochemistry variables. All laboratory tests were conducted in the hospital laboratory using standard procedures.

### Independent variables

The patients’ fasting blood glucose (FBG) levels were measured in the hospital laboratory. Blood glucose levels were defined as follows (1): slightly increased: FBG ≤11.1 mmol/L (2); moderately increased: FBG 11.2–16.6 mmol/L and (3) severely increased: FBG >16.6 mmol/L.

### Health-related behaviors

Five self-reported health behaviors, current smoking status, alcohol use, physical activity, sleep duration, and sleep quality, were used as outcomes and operationally defined. Smoking was defined as smoking at least one cigarette per day for ≥6 months (23) and differentiated into current smoking versus non-smoking. Alcohol use was defined as consuming at least one glass of alcohol in the past month (24). Physical activity was determined by yes/no responses to the question, “do you engage in physical activity at least 30 minutes at work and/or during leisure time more than four days a week” (25). Sleep duration was identified by two questions, “how early do you usually go to sleep at night and how early do you usually wake up” (26). Sleep quality was determined by poor/moderate/good responses to the question “how do you rate your sleep quality” (21).

### Physical health

Physical health characteristics included body mass index (BMI), according to the formula: BMI = weight (kg)/height (m^2^). New cases, mean no history of diabetes and newly diagnosed cases of type 2 diabetes (yes vs. no), other chronic diseases (yes vs. no), T2DM complications (yes vs. no), take hypoglycemic drugs and disease duration (continuous data).

### Demographic variables

Age, marital status (married or single), residence place (rural or urban), occupation, and family income (average monthly family income self-reported in the local currency (RMB)) were considered demographic information.

### Statistical analyses

We expressed continuous and categorical variables as mean (SD) and percentages (%). Comparisons were made between frequencies and means using t-tests and Chi-square tests. Multiple mixed-effect linear regression was used to examine the association between blood glucose levels and lipids and lipid ratio parameters. In the multiple mixed-effect linear regression model, the independents and dependent variables have a linear relationship, and the dependent variable must be continuous and at least interval-scale. Besides, the residuals follow the normal probability distribution and are independent. In this study, three separate models were used to control for each covariate: 1) adjusted for the demographic variables, 2) adjusted for demographics and health-related behaviors, and 3) adjusted for demographics, health-related behaviors, and physical health. Gender was fitted as a random intercept model. The regression models were shown with beta coefficients and 95% confidence intervals. All the analyses were performed using STATA software 14.0.

## Results

### Demographic characteristics of the participants

The patients had an average age of 58.6 (SD=12.1) years. More than half (54.8%) were male, 40.1% were farmers, and nearly one-fifth were illiterate (**Table 1**). A majority of the participants (63.8%) had a low blood glucose level, and 25.4% and 10.8% had moderate and high blood glucose levels, respectively. The female patients had less education, lower current smoking and alcohol use, and better sleep conditions than the males (*P<*0.05). Female patients also had higher HDL-C and LDL-C, and TG/HDL-C ratios than the male patients.

**Table 1:** Demographic characteristics of participants.

	Total n = 1747	male n = 958	female # n = 789	*P* value
Age, mean (SD), years	58.6 (12.1)	55.9 (12.4)	61.8 (11.0)	<0.001
Marital status				<0.001
Married, n (%)	1630 (93.3)	923 (96.3)	707 (89.6)	
Unmarried, n (%)	117 (6.7)	35 (3.7)	82 (10.4)	
Residence				<0.001
Urban, n (%)	1095 (62.7)	657 (68.6)	438 (55.5)	
Rural, n (%)	652 (37.7)	301 (31.4)	351(44.5)	
Occupation, farmer	701 (40.1)	301 (31.4)	400 (50.7)	<0.001
Education attainment				<0.001
Illiterate, n (%)	348 (19.9)	71 (7.4)	277(35.1)	
Primary, n (%)	392 (22.4)	184(19.2)	208 (26.4)	
Junior and Senior, n (%)	616 (35.3)	415 (43.3)	201 (25.5)	
College degree or above, n (%)	391 (22.4)	288 (30.1)	103 (13.1)	
Family income				<0.001
<2000, n (%)	680 (38.9)	309 (32.2)	371 (47.0)	
>2000, n (%)	1067 (61.1)	649 (67.8)	418 (53.0)	
Smoking				<0.001
Yes, n (%)	393 (22.5)	381 (39.8)	12 (1.5)	
No, n (%)	1354 (77.5)	577 (60.2)	777 (98.5)	
Alcohol use				<0.001
Yes, n (%)	365 (20.9)	345 (36.0)	20 (2.5)	
No, n (%)	1382 (79.1)	613 (64.0)	769 (97.5)	
Physical exercise				0.842
Yes, n (%)	1169 (66.9)	643 (67.1)	526 (66.7)	
No, n (%)	578 (33.1)	315 (32.9)	263 (33.3)	
Sleep duration, mean (SD), hour	8.2 (1.2)	8.0 (1.2)	8.1 (1.4)	<0.001
Sleep quality, poor, n (%)	393 (22.5)	167 (17.4)	226 (28.6)	<0.001
BMI, mean (SD), kg/m^2^	1.8 (0.7)	1.8 (0.7)	1.7 (0.7)	0.001
New cases, yes, n (%)	273 (15.6)	180 (18.8)	93 (11.8)	<0.001
Other chronic diseases, yes, n (%)	1149 (65.8)	592 (61.8)	557 (70.6)	<0.001
Disease duration, mean (SD), years	8.3 (7.6)	7.8 (7.4)	8.1 (1.1)	0.002
T2DM complications, yes, n (%)	1048 (60.0)	577 (60.2)	471 (59.7)	0.821
take hypoglycemic drugs, yes, n (%)	1457(83.4)	797 (83.2)	660 (83.7)	0.799
Blood glucose level				0.166
slightly increased, n (%)	1114 (63.8)	600 (62.6)	514 (65.1)	
moderately increased, n (%)	444 (25.4)	260 (27.1)	184 (23.3)	
severely increased, n (%)	189 (10.8)	98 (10.2)	91 (11.5)	
TG, mean (SD), mmol/L	2.2 (2.1)	2.2 (2.2)	2.2 (1.8)	0.532
TC, M(Q), mmol/L	4.5 (3.7,5.0)	4.3 (3.6,4.8)	4.6 (3.9,5.2)	0.683
HDL-C, mean (SD), mmol/L	1.1 (0.3)	1.0 (0.3)	1.1 (0.3)	<0.001
LDL-C, mean (SD), mmol/L	2.6 (0.9)	2.5 (0.8)	2.8 (0.9)	<0.001
TG/HDL-C, M(Q)	1.7 (1.0,2.4)	1.7 (1.0,2.5)	1.7 (1.1,2.3)	0.008
TC/HDL-C, M(Q)	4.2 (3.4,4.9)	4.2 (3.4,5.0)	4.1 (3.4,4.7)	0.209
LDL-C/HDL-C, mean (SD)	2.5 (0.9)	2.6 (1.0)	2.5 (0.9)	0.297

SD, standard deviation; BMI, body mass index; T2DM, type 2 diabetes mellitus; TG, triglyceride; TC, total cholesterol; HDL-C, high-density lipoprotein cholesterol; LDL-C, low-density lipoprotein cholesterol.

### Bivariate regression model

**Figure 1:**
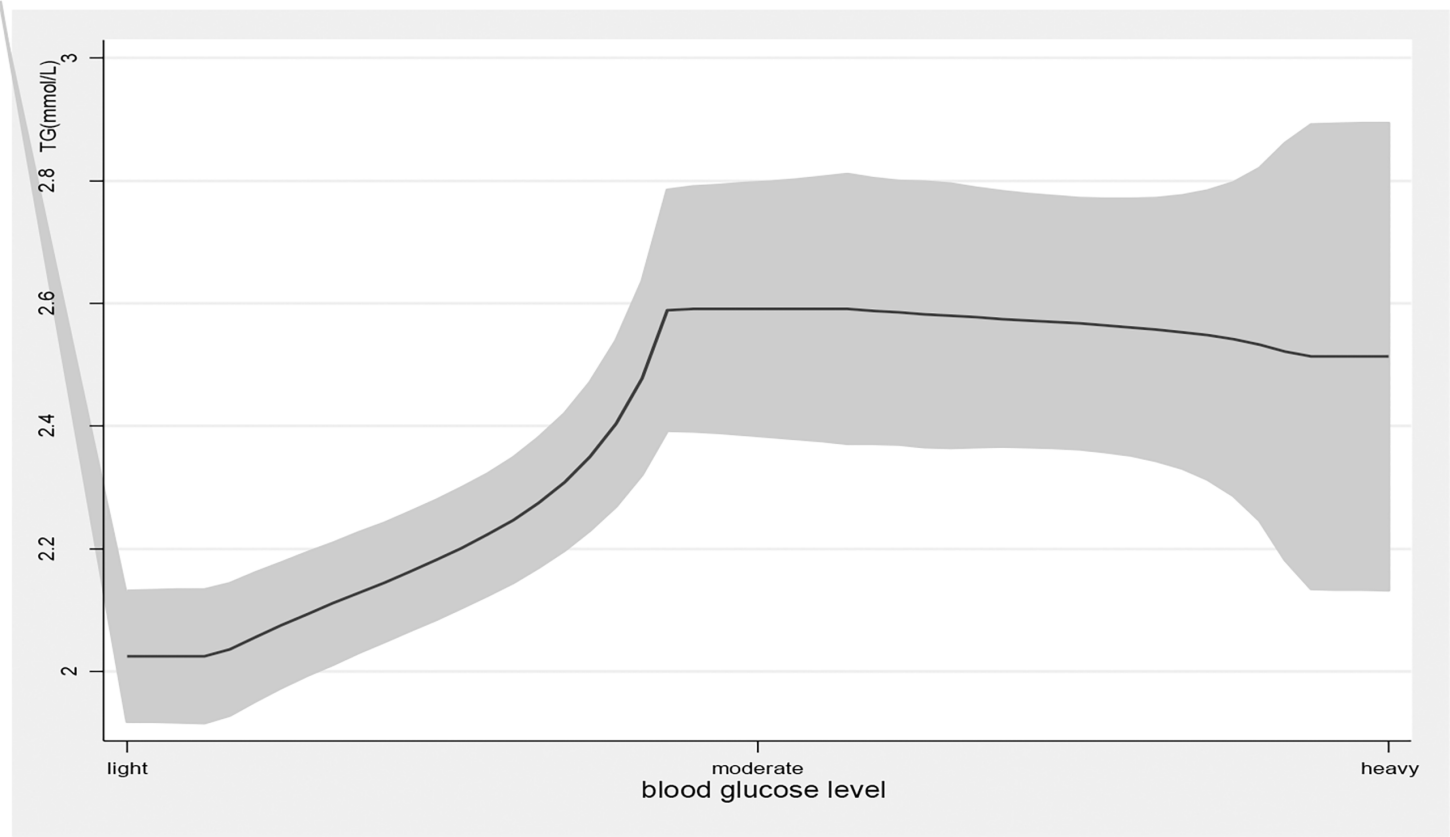
the level of TG with the change of blood glucose level. with the high level of blood glucose, the TG level was higher. line represent the value of TG, the gray confidence intervals represent the 95% confidence intervals of TG.

Age, education attainment, current smoking, alcohol use, physical activity, BMI, new cases, other chronic diseases, and disease duration were associated with TG levels; current smoking, physical exercise, BMI, disease duration, T2DM complications, and taking medicine were associated with HDL levels; family income, physical exercise, new cases, and disease duration were associated with LDL levels; and age, education attainment, current smoking, alcohol use, physical exercise, BMI, new cases, disease duration, and T2DM complications were associated with TG/HDL-C (**Table 2**). Blood glucose levels were associated with high TG levels (β=0.34, 95% *CI*: (0.20, 0.48), *p<*0.01); 1 mmol/L level increase in blood glucose correlated with a 0.34 mmol/L increase in TG. Blood glucose levels were also associated with high levels of LDL (β=0.08, 95% *CI*: (0.02, 0.14), *p<*0.01) and elevated TG/HDL-C (β=0.31, 95% *CI*: (0.13, 0.49), *p<*0.01) and LDL-C/HDL-C (β=0.13, 95% *CI*: (0.06, 0.20), *p<*0.01) ratios. As blood glucose levels increased, there was a significant increase in TG and LDL-C levels and TG/HDL-C and LDL-C/HDL-C ratios (**Figures 1**, **4**).

**Table 2:** Binary regression model.

	TG *β* (95%*CI*)	TC *β* (95%*CI*)	HDL *β* (95%*CI*)	LDL *β* (95%*CI*)	TG/HDL-C *β* (95%*CI*)	TC/HDL-C *β* (95%*CI*)	LDL-C/HDL-C *β* (95%*CI*)
Age	-0.02 (-0.03,-0.01)**	0.02(-0.02,0.07)	0.001(0.00,0.002)	-0.00 (-0.00,0.00)	-0.03 (-0.04,-0.02)**	0.02 (-0.03,0.06)	-0.004 (-0.008,-0.001)*
Marital status	0.08(-0.31,0.47)	0.31(-1.90,2.52)	-0.03 (-0.08,0.03)	-0.10 (-0.27,0.06)	0.14 (-0.36,0.63)	0.40 (-1.84,2.64)	-0.06 (-0.24,0.13)
Residence	-0.02(-0.22,0.19)	-0.85(-1.99,0.29)	-0.02 (-0.05,0.01)	-0.01(-0.09,0.08)	0.17 (-0.08,0.43)	-0.73 (-1.89,0.43)	0.06 (-0.04,0.15)
Occupation	0.08(-0.12,0.28)	0.79(-0.34,1.92)	0.01 (-0.01,0.04)	-0.02 (-0.10,0.07)	-0.10 (-0.35,0.15)	0.68 (-0.46,1.82)	-0.07 (-0.16,0.02)
Education attainment	0.09 (0.01,0.19) *	-0.16(-0.69,0.37)	-0.01 (-0.02,0.00)	0.01 (-0.03,0.04)	0.17 (0.05,0.29)**	-0.07 (-0.61,0.47)	0.06 (0.01,0.10)*
Family income	0.04(-0.03,0.12)	-0.01(-0.44,0.43)	-0.01 (-0.02,0.00)	0.04 (0.01,0.07)**	0.06 (-0.04,0.16)	0.04 (-0.40,0.49)	0.07 (0.03,0.10)**
Smoking	0.52 (0.28, 0.75)**	-0.37(-1.69,0.96)	-0.06 (-0.09,-0.02)**	-0.02 (-0.11,0.08)	0.83 (0.54,1.12)**	-0.02 (-1.37,1.32)	0.16 (0.05,0.27)**
Alcohol use	0.51 (0.27,0.75)**	-0.30(-1.66,1.06)	-0.03 (-0.06,0.01)	-0.01 (-0.11,0.09)	0.49 (0.18,0.79)**	-0.22 (-1.59,1.16)	0.05 (-0.06,0.16)
Physical exercise	-0.18 (-0.47,-0.08)**	0.65(-0.47,1.77)	0.05 (0.02,0.08)**	-0.14 (-0.22,-0.06)**	-0.48 (-0.73,-0.23)**	0.41 (-0.73,1.54)	-0.26 (-0.35,-0.17)**
Sleep duration	-0.05(-0.13,0.03)	0.17(-0.30,0.65)	0.01 (-0.01,0.02)	-0.01 (-0.05,0.02)	-0.08 (-0.18,0.03)	0.14 (-0.34,0.62)	-0.03 (-0.07,0.01)
Sleep quality	-0.12(-0.25,0.01)	0.01(-0.74,0.76)	0.01 (-0.01,0.03)	0.04 (-0.02,0.09)	-0.05 (-0.21,0.129)	0.02 (-0.74,0.78)	0.03 (-0.03,0.09)
BMI	0.37(0.23,0.50)**	0.51(-13.0,16.1)	-0.03 (-0.05,-0.01)**	0.02 (-0.04,0.07)	0.47 (0.30,0.63)**	0.25 (-0.52,1.03)	0.08 (0.01,0.14)*
New cases	0.48 (0.21,0.74)**	-0.06(-1.58,1.47)	-0.02 (-0.05,0.02)	0.15 (0.04,0.26)**	0.38 (0.04,0.72)*	-0.05 (-1.59,1.49)	0.16 (0.03,0.29)*
Other chronic diseases	0.30 (0.09,0.50)**	0.48(-0.68,1.65)	-0.01 (-0.04,-0.02)	-0.04 (-0.13,0.04)	0.13 (-0.13,0.39)	0.41 (-0.77,1.59)	-0.07 (-0.17,0.02)
Disease duration	-0.03 (-0.05,-0.02)**	0.86(-1.62,3.34)	0.002(0.001,0.004)*	-0.008 (-0.013,-0.002)**	-0.03 (-0.05,-0.02)**	-0.01 (-0.09,0.06)	-0.01 (-0.02,-0.005)**
T2DM complications	-0.14(-0.34,0.06)	-0.80(-1.93,0.33)	0.03 (0.01,0.06)*	0.01 (-0.07,0.09)	-0.30 (-0.54,-0.04)*	-0.95 (-2.09,0.20)	-0.04 (-0.13,0.05)
take hypoglycemic drugs	-0.07(-0.33,0.19)	0.32(-1.19,1.84)	-0.03 (-0.06,0.10)	-0.15 (-0.26,-0.04)*	-0.06 (-0.39,0.26)	0.41 (-1.12,1.95)	-0.13 (-0.26,-0.01)*
Blood glucose level	0.34 (0.20,0.48)**	0.42(-0.39,1.23)	-0.01 (-0.03,0.01)	0.08 (0.02,0.14)**	0.31 (0.13,0.49)**	0.50 (-0.32,1.32)	0.13 (0.06,0.20)**

**:P < 0.01 *p < 0.05 β, beta; BMI, body mass index; T2DM, type 2 diabetes mellitus.

**Figure 2:**
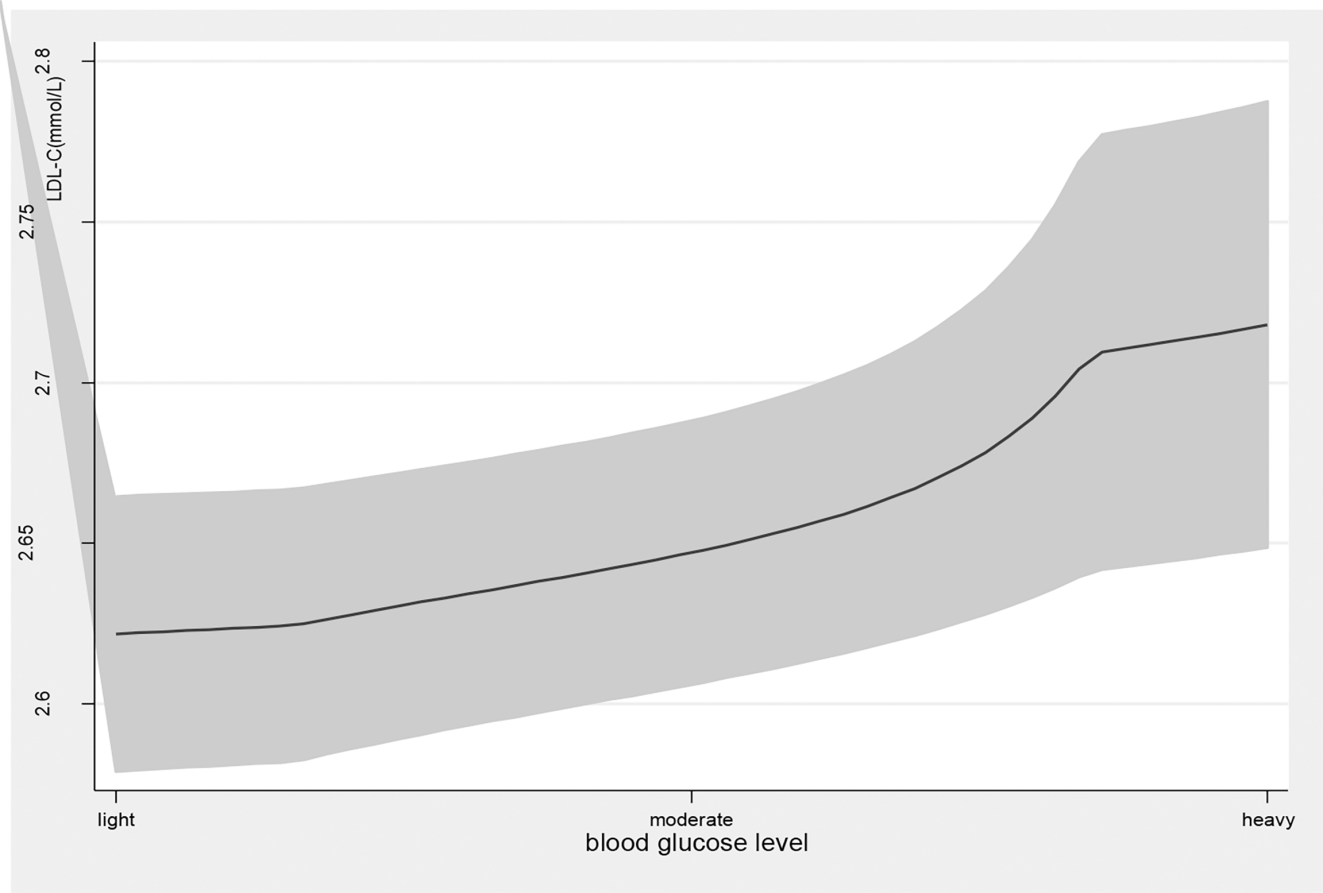
the level of LDL-C with the change of blood glucose level. with the high level of blood glucose, the LDL-C level was higher. line represent the value of LDL-C, the gray confidence intervals represent the 95% confidence intervals of LDL-C.

**Figure 3:**
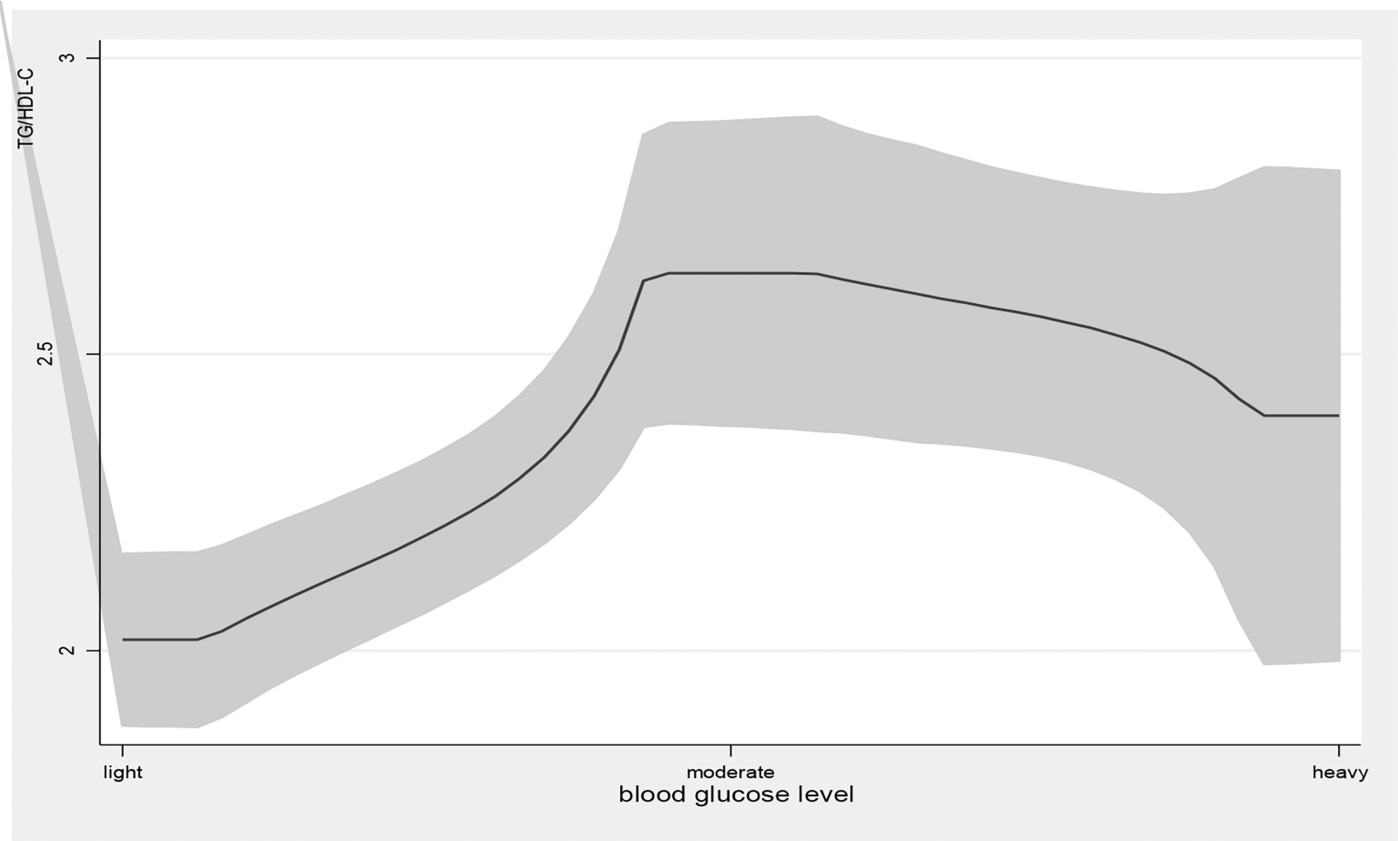
the level of TG/HDL-C with the change of blood glucose level. with the high level of blood glucose, the TG/HDL-C level was higher. line represent the value of TG/HDL-C, the gray confidence intervals represent the 95% confidence intervals of TG/HDL-C.

**Figure 4:**
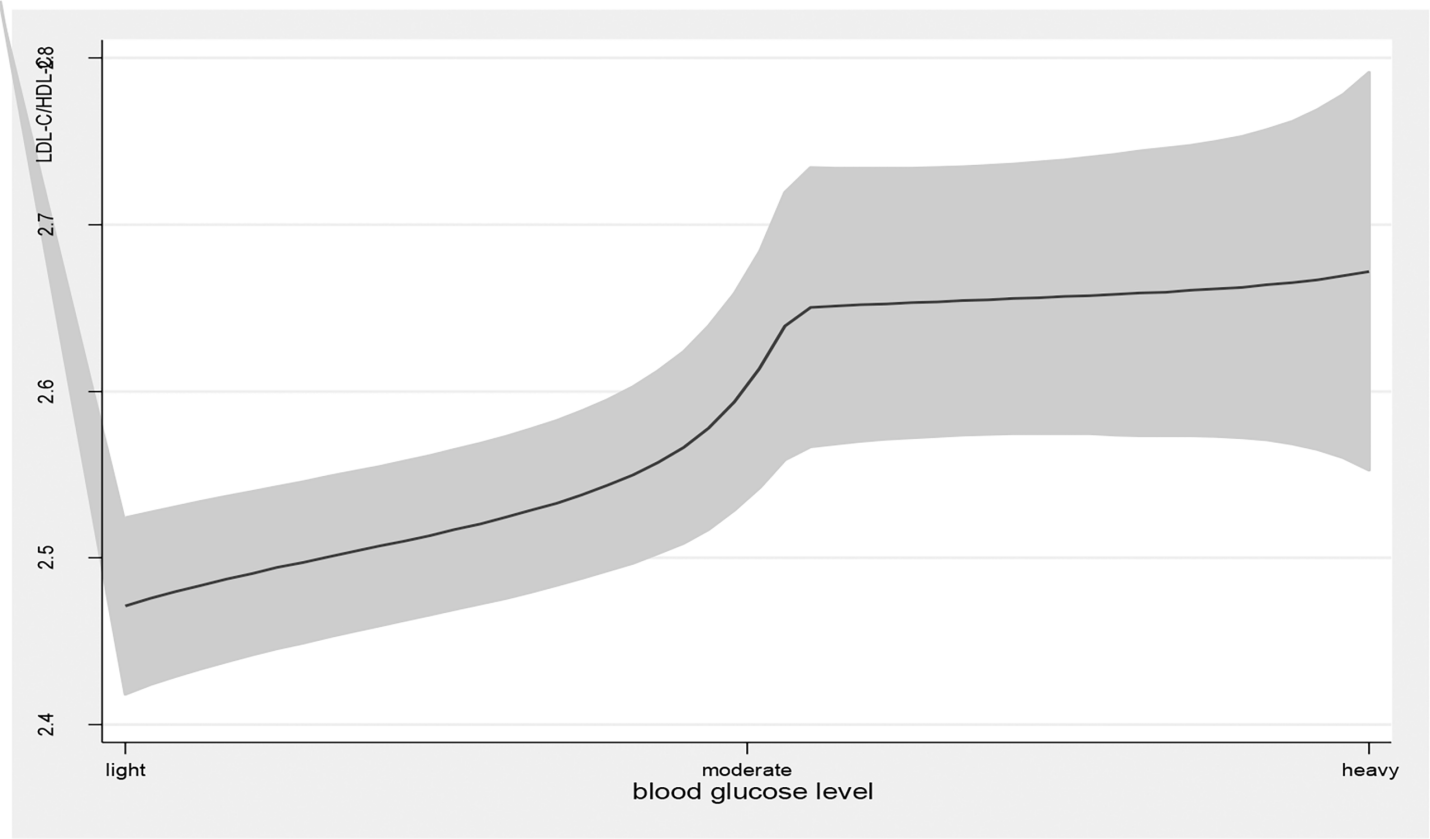
the level of LDL-C/HDL-C with the change of blood glucose level. with the high level of blood glucose, the LDL-C/HDL-C level was higher. line represent the value of LDL-C/HDL-C, the gray confidence intervals represent the 95% confidence intervals of LDL-C/HDL-C.

### Multiple mixed-effect linear regression models measuring the association between blood glucose levels and lipids and lipid ratios

Model 1 found a positive association between blood glucose levels and TG (β=0.31, 95% *CI*: (0.17, 0.45), *p<*0.01) and LDL-C (β=0.08, 95% *CI*: (0.02, 0.13), *p<*0.01) levels and TG/HDL-C (β=0.28, 95% *CI*: (0.09, 0.46), *p<*0.01) and LDL-C/HDL-C (β=0.11, 95% *CI*: (0.04, 0.18), *p<*0.01) ratios after controlling for the demographic variables (**Table 3**). The association persevered when controlling for health-related behaviors (Model 2) and physical health variables (Model 3). All three models established no significant association between blood glucose levels and TC or HDL-C.

**Table 3:** Multiple mixed-effect linear regression model.

	TG *β* (95%*CI*)	TC *β* (95%*CI*)	HDL *β* (95%*CI*)	LDL *β* (95%*CI*)	TG/HDL-C *β* (95%*CI*)	TC/HDL-C *β* (95%*CI*)	LDL-C/HDL-C *β* (95%*CI*)
Model 1	0.31 (0.17,0.45)**	0.46 (-0.37,1.28)	-0.01 (-0.02,0.02)	0.08 (0.02,0.13)*	0.28 (0.09,0.46)**	0.51 (-0.32,1.35)	0.11 (0.04,0.18)**
Model 2	0.30 (0.16,0.45)**	0.47 (-0.36,1.30)	-0.01 (-0.02,0.02)	0.07 (0.01,0.13)*	0.27 (0.09,0.45)**	0.53 (-0.31,1.37)	0.11 (0.04,0.18)**
Model 3	0.32 (0.18,0.46)**	0.52 (-0.31,1.35)	-0.01 (-0.02,0.01)	0.07 (0.02,0.13)*	0.30 (0.12,0.48)**	0.59 (-0.25,1.43)	0.12 (0.05,0.18)**

Model 1: blood glucose level + demographic characteristics (age, marital status, residence, occupation, education attainment, economic condition).

Model 2: Model 1+health-related behaviors (smoking, alcohol use, physical exercise, sleep duration, sleep quality).

Model 3: Model 2+physical health (BMI, new cases, other chronic diseases, disease duration, T2DM complications, take hypoglycemic drugs).

**:P < 0.01 *p < 0.05 β, beta.

## Discussion

The findings from this study add to the growing body of evidence supporting the association between blood glucose levels and serum lipid levels, and lipid ratios. Furthermore, to our knowledge, this is one of the first studies that examined the relationships between blood glucose levels and lipid ratios in a random cohort of Chinese T2DM patients. Blood glucose levels were positively associated with TG and LDL-C levels and TG/HDL-C and LDL-C/HDL-C ratios after controlling for possible confounders with a mixed-effect linear regression model. This suggests that those with higher blood glucose levels have a higher level of harmful lipids (TG, LDL, TG/HDL-C, LDL-C/HDL-C) that could increase their risk for T2DM complications.

The results of this study were supported by a previous study showing that blood glucose levels correlated positively with TG and LDL-C, which are linked to a higher risk of obesity and cardiovascular disease (27). In the context of T2DM, reasonable glycemic control could help improve lipid profiles. A previous study reported that short-term intensive glycemic control could significantly reduce T2DM-related triglyceride levels (28). According to another study, controlling blood lipid levels is one of the most significant factors influencing blood glucose control in patients with T2DM (29). In addition, blood glucose is associated with an increased risk of obesity (a determinant of lipid or lipid ratio parameters) (30).

It has been found that blood glucose levels are positively correlated with TG and LDL-C levels, while others have found no association (31, 32). Recently, a study reported that fasting plasma glucose is related to HDL and TC but not to LDL or TG (18). Meanwhile, a survey conducted in Eastern Sudan found that poor glycemic control was not related to TG but was related to elevated TC (32). Substantial heterogeneity in the study population, design, and variable definition, or an inadequate adjustment for medical confounding may explain the discrepancy. In addition, several factors affect the blood glucose levels and lipid profiles of people with T2DM. For example, diet is a significant factor affecting both (33), and particular eating patterns may contribute to different relationships between blood glucose levels and lipid profiles (34, 35).

Our findings suggest that blood glucose levels are positively associated with TG/HDL-C and LDL-C/HDL-C ratios. A ratio of lipid profile is more valuable than participant lipid values because it more accurately represents the complex interactions that affect the metabolism of lipoproteins (36). Researchers have indicated that the lipid ratio represents the ratio of atherogenic to antiatherogenic lipoproteins (37). It is assumed that individuals with high blood glucose levels have a high TC/HDL‐C ratio and are more likely to suffer stroke or atherosclerosis.

### Strengths and limitations

As a strength of this study, it uses a patient-based and multi-stage random sampling design, in addition to a large sample size with lipid and lipid ratio measurements. However, there are several limitations. First, causal relationships between blood glucose and lipids cannot be determined because of its cross-sectional design. Second, despite controlling for numerous covariates, residual confounders could not be completely eliminated due to unmeasured factors. Especially, here lack of the information about the dosage of hypoglycemic drugs, so it may affect our results. Third, the study included patients from one province of China, so caution should be used when applying these findings to other regions of China or foreign countries.

## Conclusion

A correlation between blood glucose levels and serum lipids or lipid ratios has been established in this study. Blood glucose levels were positively associated with TG and LDL-C levels and elevated TG/HDL-C and LDL-C/HDL-C ratios.

## Data availability statement

The original contributions presented in the study are included in the article/**Supplementary Material**. Further inquiries can be directed to the corresponding author.

## Ethics statement

The studies involving human participants were reviewed and approved by The Institutional Review Board of the Yinchuan Hospital of Traditional Chinese Medicine. The patients/participants provided their written informed consent to participate in this study.

## Author contributions

WLQ and NY designed research; YN, ZM, PRP, DYQ conducted research; WLQ and YN analyzed data; WLQ, NY and YN wrote the paper; NY had primary responsibility for final content. All authors contributed to the article and approved the submitted version.

## Funding

This work was supported by the Ningxia Natural Science Foundation (2022AAC05058).

## Acknowledgments

We would like to express our sincere gratitude to the participants for their effort in this research. We also thank the investigators for their hard-working interviewing and data checking.

## Conflict of interest

The authors declare that the research was conducted in the absence of any commercial or financial relationships that could be construed as a potential conflict of interest.

## Publisher’s note

All claims expressed in this article are solely those of the authors and do not necessarily represent those of their affiliated organizations, or those of the publisher, the editors and the reviewers. Any product that may be evaluated in this article, or claim that may be made by its manufacturer, is not guaranteed or endorsed by the publisher.

## Abbreviations

T2DM, type 2 diabetes mellitus; TC, total cholesterol; TG, triglyceride; LDL‐C, low‐density lipoprotein cholesterol; HDL‐C, high‐density lipoprotein cholesterol; PPS, probability proportionate to size; FBG, fasting blood glucose; BMI, body mass index.
